# Improving In-Situ Estimation of Soil Profile Properties Using a Multi-Sensor Probe

**DOI:** 10.3390/s19051011

**Published:** 2019-02-27

**Authors:** Xiaoshuai Pei, Kenneth A. Sudduth, Kristen S. Veum, Minzan Li

**Affiliations:** 1 Key Laboratory of Modern Precision Agriculture System Integration Research-Ministry of Education, China Agricultural University, Beijing 100083, China; xiaoshuaipei@163.com (X.P.); limz@cau.edu.cn (M.L.); 2 USDA-ARS Cropping Systems and Water Quality Research Unit, Columbia, MO 65211, USA; Kristen.Veum@ars.usda.gov

**Keywords:** diffuse reflectance spectroscopy, precision agriculture, profile soil properties, proximal soil sensing, in-situ sensing

## Abstract

Optical diffuse reflectance spectroscopy (DRS) has been used for estimating soil physical and chemical properties in the laboratory. In-situ DRS measurements offer the potential for rapid, reliable, non-destructive, and low cost measurement of soil properties in the field. In this study, conducted on two central Missouri fields in 2016, a commercial soil profile instrument, the Veris P4000, acquired visible and near-infrared (VNIR) spectra (343–2222 nm), apparent electrical conductivity (EC_a_), cone index (CI) penetrometer readings, and depth data, simultaneously to a 1 m depth using a vertical probe. Simultaneously, soil core samples were obtained and soil properties were measured in the laboratory. Soil properties were estimated using VNIR spectra alone and in combination with depth, EC_a_, and CI (DECS). Estimated soil properties included soil organic carbon (SOC), total nitrogen (TN), moisture, soil texture (clay, silt, and sand), cation exchange capacity (CEC), calcium (Ca), magnesium (Mg), potassium (K), and pH. Multiple preprocessing techniques and calibration methods were applied to the spectral data and evaluated. Calibration methods included partial least squares regression (PLSR), neural networks, regression trees, and random forests. For most soil properties, the best model performance was obtained with the combination of preprocessing with a Gaussian smoothing filter and analysis by PLSR. In addition, DECS improved estimation of silt, sand, CEC, Ca, and Mg over VNIR spectra alone; however, the improvement was more than 5% only for Ca. Finally, differences in estimation accuracy were observed between the two fields despite them having similar soils, with one field demonstrating better results for all soil properties except silt. Overall, this study demonstrates the potential for in-situ estimation of profile soil properties using a multi-sensor approach, and provides suggestions regarding the best combination of sensors, preprocessing, and modeling techniques for in-situ estimation of profile soil properties.

## 1. Introduction

Traditional agriculture applies uniform management to fields without considering the spatial heterogeneity of soil properties and plant growth. This contributes to potential over-application of chemical inputs such as fertilizer, pesticides, and herbicides, leading to increased environmental risk. In contrast, precision agriculture aims to manage at the field scale and apply inputs according to the needs of each area within a field. Thus, precision agriculture has the potential to improve crop production, prevent excess application of chemical inputs, reduce expenses, and reduce environmental impacts. To reach this goal, site-specific soil properties that affect plant growth and crop production need to be measured to provide a basis for precision agriculture management. Measuring multiple soil properties at a high temporal and/or spatial frequency is time consuming and costly, using conventional methods. Therefore, alternative approaches are needed to achieve high resolution, field scale information for producers.

Diffuse reflectance spectroscopy (DRS) is a promising, fast, nondestructive soil sensing technique that does not require laboratory soil analysis other than for development of calibration equations [1]. Many research projects have estimated soil physical and chemical properties using DRS in the visible (400 to 700 nm), near-infrared (NIR; 700–2500 nm), or combined VNIR wavelength ranges [2]. With acceptable accuracy, diffuse reflectance spectroscopy could replace standard laboratory measurement methods at a reasonable cost in some cases. Furthermore, in-situ DRS measurements can provide an additional level of efficiency by removing the need to collect soil and transport it to the laboratory. In-situ DRS instruments can provide results immediately if suitable calibrations have already been developed and successfully applied in field settings [3,4,5]. Many soil properties have been successfully estimated using DRS. For example, total carbon has been estimated with R^2^ ranging from 0.73 to 0.95 [3,6,7,8,9]. Similarly, many studies have successfully estimated soil organic carbon (SOC), cation exchange capacity (CEC), calcium (Ca), potassium (K), texture (clay, silt and sand fractions), magnesium (Mg), pH, and other soil properties with DRS [6,10,11,12,13,14,15].

Studies estimating soil properties using DRS, including those described above, have primarily focused on surface soils, typically in the top 5–30 cm of the profile. In general, agricultural management and associated soil measurements focus on this surface layer that represents the interface between the soil and the atmosphere and is critical in plant growth and development. However, soil properties deeper in the profile are controlling factors for many aspects of soil function, including carbon storage and nutrient cycling (e.g., [16,17]). Therefore, profile DRS data and other profile soil information are potentially useful for agricultural management. Previous laboratory studies have demonstrated successful estimation of profile soil properties with DRS. For example, Dalal and Henry [18] estimated profile SOC, total nitrogen (TN), and soil moisture with R^2^ > 0.84. Others used NIR DRS to estimate profile moisture (R^2^ = 0.87) and SOC (R^2^ > 0.77) in the laboratory [19], and to estimate several profile soil properties, including clay, Ca, CEC, and SOC with R^2^ ≥ 0.80 [20]. Another approach has been in-field spectrometry on intact, extracted soil cores [4], which was reported to successfully predict SOC at depth resolutions of up to 1 cm [21,22]. 

Acquisition of in-situ, profile soil information has been investigated by several groups, as it offers many potential benefits over laboratory DRS analysis of profile samples in terms of efficiency, timeliness, and expense. A penetrometer foreoptic developed for a prototype NIR spectrophotometer estimated soil moisture with R^2^ = 0.90 [23]. In field studies, prototype penetrometer foreoptics coupled to commercial spectrometers estimated clay content with 20–25% greater error compared to laboratory spectra on dried and sieved samples [24,25]. A commercial penetrometer instrument, the Veris P4000 (Veris Technologies, Salina, Kansas, USA) can be deployed in the field for profile VNIR DRS data collection. Data from this instrument has been used to estimate multiple soil properties. In one study, the cross-validation R^2^ of bulk density (BD), SOC, moisture, clay, silt, and sand were found to be 0.32, 0.67, 0.40, 0.65, 0.61, and 0.38, respectively [26]. In another study, texture and soil organic matter (SOM) were estimated with errors of ~6% for clay and silt, 10 to 11% for sand, and 0.3 to 0.5% for SOM [27]. Soil organic carbon estimates from in-situ Veris P4000 data were less accurate (R^2^ from 0.78 to 0.90) than estimates from P4000 data obtained in the laboratory on dry soil samples (R^2^ from 0.93 to 0.96) [28]. Veum et al. [29] reported similar results, with the root mean square error (RMSE) of prediction smaller (0.19%) for SOC estimates based on laboratory DRS than for estimates based on in-situ DRS (0.26%).

Soil property estimation with VNIR DRS requires the application of a multivariate calibration algorithm, often after the spectral data have been subjected to one or more types of mathematical pretreatment. No general consensus on the best spectral pretreatments has emerged in the literature [1]; rather the need to examine multiple pretreatments and combinations in a “trial-and-error” approach has been reported, both for different datasets and for different soil properties within the same dataset [30]. In some studies, little difference in calibration results has been reported across a range of pretreatments [31], but that is not always the case. Likewise, no single calibration algorithm has consistently provided the best results. In some cases, neural networks and machine learning algorithms have been reported as better than the more commonly used partial least squares regression (PLSR) [32]. In other studies, machine learning algorithms and PLSR have provided similar results [31,33]. Most evaluations of pretreatments and calibration algorithms have been done using laboratory DRS—few studies have done this evaluation for in-situ DRS. Thus, there is a need for further investigation of pretreatments and calibration algorithms applied to in-situ sensing.

Many studies have illustrated that VNIR spectra are sensitive to variable environmental conditions in the field, such as changes in soil structure, temperature, and moisture [11,34,35,36]. Specifically, OH bands from soil moisture are known to mask important spectral features produced by SOC and other soil properties [3], meaning that lab-based results on dry soil are often more accurate than in-situ results with field-moist soil. Thus, successful in-situ or in-field, profile estimation of soil properties often requires balancing a reduction in accuracy compared to laboratory analysis with improvements in operational efficiencies. One option for improving these measurements may be to combine data from auxiliary sensors with VNIR DRS data. For example, an in-field core-scanning system that included gamma-ray attenuation and digital imaging along with VNIR DRS was used to estimate multiple soil profile properties [37]. The Veris P4000 can measure apparent soil electrical conductivity (EC_a_), and penetrometer cone index (CI), along with the depth of measurement. Adding these data to the calibration has been investigated for their potential to improve the estimation of the physical and chemical properties of soil profile [26,27]. Soil EC_a_ reflects numerous soil physical and chemical attributes such as texture, mineralogy, CEC, and moisture [38,39,40]. Cone index, defined as the force per unit base area required to push a penetrometer through a specified increment of soil [41,42], is affected by soil compaction, soil bulk density, texture, and moisture [43,44,45]. 

The primary objective of this study was to estimate multiple soil profile properties using in-situ sensor data obtained using the Veris P4000 instrument: VNIR reflectance, EC_a_, CI, and depth. The target soil properties included SOC, TN, soil moisture, soil texture (clay, silt, and sand), CEC, Ca, Mg, K, and pH. The specific objectives were to evaluate and compare estimation accuracy across multiple soil properties for:

- Ten different spectral preprocessing techniques.
- Four calibration methods: PLSR, neural networks, regression trees, and random forests.
- All four sensors in combination compared to VNIR spectra alone.
- Single-field calibrations compared to those developed for multiple fields.

## 2. Materials and Methods

### 2.1. Study Fields

Sensor data and soil samples were obtained in March 2016 at Field 1 (F1, 36 ha) and Field 3 (F3, 20 ha), two long-term research sites [46] located within 3 km of each other, near Centralia (39.230° N, 92.117° W), in central Missouri, USA. Corn, soybean, and wheat were cropped in F1 under no-tillage, while corn and soybean were cropped in F3 with tillage. Spring tillage occurred in F3 to an approximate 13 cm depth one week prior to sensing, and the tilled surface soil was dry and loose. Both F1 and F3 were located on claypan soils, which are distinguished by a greater than 50% increase in clay from topsoil to subsoil horizons. Measured topsoil depth above the claypan (depth to the first Bt horizon) in these fields ranged from less than 0.1 m to greater than 1 m [38]. On claypan soils, variation in the topsoil depth above the restrictive claypan layer leads to variation in hydrology, profile soil properties, and crop productivity. Surface textures at F1 and F3 ranged from silt loam to silty clay loam. The subsoil claypan horizon(s) were silty clay loam, silty clay, or clay, and contained as much as 50–60% clay.

### 2.2. Sensor Data Collection 

**Figure 1:**
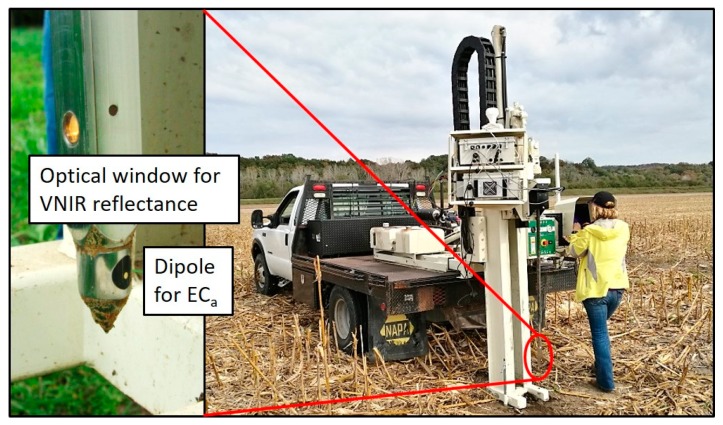
Veris P4000 instrument in field operation, collecting visible and near-infrared (VNIR) spectra, apparent soil electrical conductivity (EC_a_), and cone index (CI) penetrometer readings. A close-up view shows the probe tip, including optical window and EC_a_ dipole.

The Veris P4000 probe included a halogen light source, sapphire window, and spectrometer to collect profile VNIR absorbance (i.e., log_10_[1/reflectance]) spectra (343–2222 nm), dipole contacts for EC_a_ (mS·m^−1^) data collection, and a load cell which quantified CI (kPa) by measuring the insertion force on the conical tip of the probe (Figure 1). The resolution of the spectrometer was 6 nm in the spectral range of 342–1023 nm, and 4 nm in the spectral range of 1070–2220 nm. Depth, EC_a_, CI, and spectra (DECS) were measured simultaneously to a depth of ~1 m. To increase the signal-to-noise ratio through averaging sensor data, probe measurements were repeated five times ~15 cm apart at each location. All sensor data were obtained at a nominal 20 Hz rate as the probe was hydraulically inserted into the soil at ~30 mm·s^−1^. Instrument output data were the mean of every 25 raw measurements, and each output measurement represented an approximately 4-cm depth increment. Spectral data were visually examined before analysis to identify any near-surface scans that may have been affected by exposure of the detector to sunlight. Any such spectra were deleted prior to analysis, as were data at wavelengths <400 nm due to their low signal-to-noise ratio.

### 2.3. Soil Sampling and Laboratory Analysis

To characterize the soil at each location, one 5 cm diameter soil core was extracted, no further than 0.5 m from any probe insertion. The soil cores were described and segmented by pedogenic horizon, homogenized with a 5 mm sieve, then stored in a cooler at 4 °C until laboratory analysis. The number of horizons per core varied from 2 to 6, with a median of 4, for a total of 148 horizon samples. In total, profile data and soil cores were obtained from 20 locations in F1 and 13 locations in F3. Soil samples were analyzed for SOC and TN using a Leco TruMac C/N combustion analyzer (LECO Corp., St. Joseph, MI, USA), following standard procedures [47]. Samples were analyzed at the University of Missouri Soil Characterization Laboratory for soil texture (clay, silt, and sand fractions; %), CEC (cmol·kg^−1^), exchangeable cations (Ca, Mg, and K; cmol·kg^−1^), and pH following established methods [48]. Soil moisture was determined gravimetrically.

Table 1 presents the descriptive statistics of the laboratory-measured soil properties. Among profile soil properties, SOC, TN, silt, and Mg varied more than the other properties, with coefficients of variation (CV) greater than 50%. In the surface soil layer, sand, Ca, Mg, and K varied the most, with CV greater than 35%. 

### 2.4. Alignment of Soil and Sensor Data

Because soil and sensor data (spectra, EC_a_, and CI) were collected at different depth increments, it was necessary to combine them to a common level of spatial (i.e., vertical) support. This was done using weighted averaging of the sensor data to match the soil samples segmented by variable thickness horizons from the soil cores. The weighting procedure was based on the fact that the sensor depth recorded was the final depth of the instrument at the end of the 25-scan observation period. This depth then defined the starting depth for the next observation in the probing sequence. These sensor-data depth segments varied in thickness, with an average thickness of 4.1 cm and a standard deviation of 1.4 cm. The initial starting depth for the first observation in any probe was unknown. Therefore, we chose to start at a depth of zero for the first scan, or at a depth such that the first observation represented no more than 4.0 cm of depth. Observations that fell entirely into a single soil layer were weighted by the depth increment of the observation divided by the total thickness of the layer. Where observations spanned two soil layers, the observation was partitioned into both layers based on the amount of depth represented in each layer and again divided by layer thickness. At the end of this procedure, the weighted average sensor data were merged with the corresponding soil properties. Observations with missing laboratory or sensor data were dropped, resulting in a final dataset of 148 observations for analysis. 

### 2.5. Analysis Methods

Different combinations of spectral preprocessing techniques, sensor data sources, and modeling methods were evaluated and compared to select the best combination for in-situ estimation of profile soil properties. The procedure included: (1) comparison of ten spectral preprocessing techniques for PLSR analysis of the DECS dataset; (2) using the best preprocessing technique and PLSR, comparing DECS and VNIR spectra results; (3) using DECS and the best preprocessing technique, comparison of results from four modeling methods (detailed below); and (4) comparison of calibration models developed for a single field with those developed for multiple fields. Model evaluation was based on coefficient of determination (R^2^) and RMSE calculated in the validation dataset.

One consideration in preparing the DECS dataset was whether the scalar variables (i.e., depth, EC_a_, and CI) should receive additional weighting to compensate for the effect of many variables (n = 374) in the spectral data vector. The use of this preprocessing step, known as block scaling in PLSR analysis [49], has been investigated on other datasets collected using the P4000 instrument [26,27]. In these previous studies there was no consistent relationship between the block scaling (or weighting) factor and estimation accuracy, with one reporting a significant relationship between RMSE of prediction and magnitude of the scaling factor in only two of 36 cases [26]. Therefore, block scaling was not implemented in this study.

#### 2.5.1. Spectral Preprocessing 

After screening a larger number of preprocessing techniques on a preliminary dataset, ten different techniques were applied to the spectra in Matlab 2016 (Mathworks, Inc., Natick, MA, USA) and the results were compared:

(1) Reflectance spectra (transformed from absorbance to reflectance);
(2) Absorbance spectra (the default output format of the P4000 instrument);
(3) Mean normalized spectra, smoothed with a 9-point moving average;
(4) Spectra smoothed with a 9-point moving average and then mean normalized;
(5) 30-point moving average;
(6) 30-point Lowess smoothing;
(7) 30-point Gaussian window smoothing;
(8) 30-point Exponential smoothing;
(9) Standard normal variate (SNV) transformation;
(10) SNV plus 30-point Gaussian smoothing.

Absorbance spectra, the default output of the P4000 instrument, and the standard transformation from absorbance to reflectance (techniques 2 and 1, respectively), were evaluated without further preprocessing. The remaining preprocessing techniques (3–10) were applied to the absorbance spectra, including various normalization and moving average smoothing techniques commonly used in soil DRS sensing [1,9,10,26]. Techniques 5–8 applied various smoothing windows to the non-normalized absorbance spectra. Lowess smoothing (locally weighted scatterplot smoothing) was developed to use locally weighted linear regression to smooth data [50,51]. Gaussian window smoothing applied a Gaussian-weighted moving average. In the exponential smoothing method, the moving average was exponentially weighted. Technique 9 applied the standard normal variate (SNV) transformation to remove baseline effects due to scatter and particle size [52], while technique 10 combined SNV with 30-point Gaussian smoothing.

#### 2.5.2. Calibration Methods

Based on a review of past work and preliminary analysis of a larger set of methods on a previous dataset, four calibration methods were chosen for evaluation on the DECS dataset. These were PLSR, neural networks (NN), regression trees (RT), and random forests (RF). 

Partial least squares regression is a statistical method that finds a linear regression model by projecting the dependent and independent variables to a new space. A new set of uncorrelated variables, called factors, was created to explain the variation of predictor and response variables [53]. For high dimensional data, PLSR can estimate the importance of features, and choose the optimal number of factors without overfitting. For spectral analysis, all wavelengths can be used for developing a calibration algorithm. For this study, PLSR was implemented in Unscrambler 10.4 (CAMO Inc., Oslo, Norway) with a random cross-validation method to select the optimum number of factors up to a maximum of 15. 

The NN approach was implemented with the Neural Network Fitting Tool of Matlab 2016. Neural networks are inspired by biological nervous systems and consist of simple elements, which work in parallel. As in nature, the connections between elements largely determine NN function, and can be trained to implement a particular function by adjusting the values of the connections (weights) between elements. Ideally, a NN is able to predict a particular target after proper training. In this study, the Neural Network Fitting Tool was used to build NN by modifying example code to import spectra and soil properties. Data were divided into three subsets: 70% to train the network, 15% to validate network generalization and to stop training before overfitting, and 15% to test the performance of the trained network when predicting to a new dataset. 

Regression trees and RF were implemented in IBM SPSS Modeler 18.0 (IBM Inc., Armonk, NY, USA). Standard regression methods assume a parametric relationship between the response and predictor variables (e.g., linear, quadratic). In contrast, RT construct a set of decision rules based on the predictor variables [54,55,56]. The data are recursively divided into smaller groups by binary splits based on a single predictor variable. The splits are chosen to maximize the homogeneity of the two resulting groups. The result of RT is a tree diagram with the branches determined by the splitting rules. The maximal tree is grown first, then the trees are pruned to an optimal size by techniques such as cross-validation [57]. For this study, 70% of the data were used for training and 30% for prediction. A single tree was built with a maximum tree depth of five. The tree was pruned by cross-validation to avoid overfitting.

A RF is a collection of decision trees, regression trees in this case, where a subset of the data is selected randomly for each decision tree that is part of the RF. This concept uses a combination of tree predictors, each depending on a randomly selected subset of data [58]. Specifically, the split at each node is determined using the best split of all variables in the standard trees, using internal estimates to evaluate the importance of each variable. This method is typically robust, performs well, and is user-friendly. In this study, 100 models were built with the maximum number of nodes set to 10,000. The maximum regression tree depth was ten and the maximum number of child nodes was five. As was done for RT, 70% of the data were used for training and 30% for prediction. Building was terminated when accuracy no longer improved. 

For all calibration methods, division into training and prediction sets was done randomly by sample. It has been previously suggested [59] that such division instead be done by location, i.e., keeping the samples for all depths at a given location together. We recognize that from a theoretical standpoint, our analysis methods could produce overly optimistic results in the presence of spatially correlated soil properties. However, due to lateral spatial correlation, the same could be said, although perhaps to a lesser extent, if by-location selection was employed. When the two selection procedures were compared previously, the difference between them was generally small when >50% of the samples were used for calibration [59].

To understand the differences in our dataset, we conducted a limited analysis, comparing by-sample and by-location selection when using PLSR and NN models, and finding little consistency in results. For NN analysis, by-sample results were better for 7 of 11 soil properties, while PLSR by-sample results were better in only 3 of 11 cases. Thus, our data did not strongly support the need for by-location validation set selection. This may be due in part to our depth-sampling procedure based on soil horizons rather than the fixed depth increments used in [59], which may have reduced vertical spatial correlation in our dataset. Based on these results, we proceeded with by-sample selection, recognizing the need to more rigorously investigate this question in the future.

## 3. Results and Discussion

Multiple spectral preprocessing techniques, sensor data sources, and modeling methods were evaluated and compared to select the best combination for in-situ estimation of profile soil properties. Detailed results are provided in the sections below. 

### 3.1. Comparison of Spectral Preprocessing Techniques

The complex field environment, including variable temperature and soil moisture conditions, is known to impact the accuracy of in-situ sensor measurements. Various preprocessing techniques have the potential to improve the accuracy of in-situ measurements. Here, ten different preprocessing techniques were applied to spectral data, and multiple soil properties were subsequently estimated using DECS and PLSR. Table 2 shows summary statistics for prediction R^2^ for each spectral preprocessing technique across all soil properties. Results are presented for three datasets: F1, F3, and the combination of F1 and F3. When averaged across the three datasets, the grand (i.e., overall) mean prediction R^2^ was very similar for all preprocessing techniques, ranging from 0.58 to 0.61, with the 30-point Gaussian window smoothing and SNV plus Gaussian performing the best (R^2^ = 0.61), followed by the 30-point moving average and SNV (R^2^ = 0.60). Thus, when averaged across all variables and datasets, no single pretreatment was clearly better than the other. 

The prediction statistics of individual soil properties for each spectral preprocessing technique are shown in Table 3. These results are for the combined (F1 and F3) dataset analyzed using DECS and PLSR. Absorbance spectra performed better than reflectance spectra for all soil properties except for soil moisture (R^2^ = 0.33 and 0.43, respectively) and clay content (R^2^ = 0.54 and 0.60, respectively). Across the individual soil properties, the 30-point Gaussian window smoothing technique performed the best for four of the eleven soil properties (CEC, Ca, Mg, and pH), and was not one of the two worst preprocessing techniques for any soil property. The next best technique, SNV, also performed the best for the four soil properties (SOC, TN, moisture, and sand), but was one of the two worst techniques for Ca. Generally, there was very little difference in estimation accuracy between these top two preprocessing techniques, with ΔR^2^ < 0.01 for all but two soil properties. As the (marginally) best preprocessing technique of those evaluated, the 30-point Gaussian window smoothing was selected for use in subsequent analyses.

### 3.2. Comparison of Spectra and DECS

The combined F1 and F3 DECS dataset was compared to the dataset containing only spectra to investigate if the additional information from DECS improved estimation of soil properties. It was expected that each of the auxiliary sensors might improve results for at least some of the soil properties. For example, soil properties are known to vary with depth, so the depth measurement might be important. Soil EC_a_ has been correlated with soil moisture, clay content, CEC, and other soil properties [38,39,40]. As a measure of soil penetration resistance, CI [41,42] has been found highly correlated with soil moisture, clay content, and BD [43,44,45]. Table 4 summarizes the results of this comparison. Across all soil properties, the average prediction R^2^ of spectra and DECS was 0.58 and 0.59, respectively. Overall, DECS performed slightly better than spectra for silt, sand, CEC, Ca, and Mg. However, RMSE with DECS decreased by more than 5% only for Ca compared to spectra alone. Spectra alone performed slightly better for TN, moisture, clay content, and K, while results for SOC and pH were the same with both datasets. These results are in accordance with other studies [26,27], where additional sensor data only slightly improved accuracy compared to spectra alone, or in some cases, provided less accurate estimates. It appears, at least for these soils, that additional variables do not provide much explanatory information that is not already contained within the spectra. However, the additional data contained in the DECS dataset might provide improved results for different soils, or for a dataset containing more variation in soil types than was present in this study or other previous research [26,27]. This should be a subject of future investigation. 

### 3.3. Model Calibration Methods 

**Figure 2:**
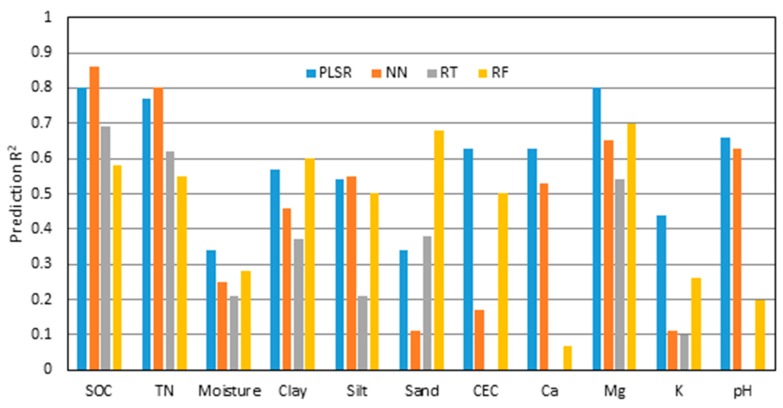
Prediction R^2^ values comparing results obtained on the combined Field 1 and Field 3 depth, EC_a_, CI and spectra (DECS) dataset using partial least squares regression (PLSR), neural networks (NN), regression trees (RT), and random forests (RF) methods. Missing bars indicate that analysis did not converge to a solution.

Multiple calibration modeling methods (i.e., PLSR, NF, RT, and RF) were compared for the estimation of soil properties. The PLSR method was either the most accurate or the second most accurate method for each of the soil properties (Figure 2). The average R^2^ values across all soil properties for PLSR, NN, RT, and RF were 0.59, 0.46, 0.39, and 0.45, respectively. When looking at individual soil properties, PLSR results were best for six of 11 (soil moisture, CEC, Ca, Mg, K, and pH). Neural network results were best for SOC, TN, and silt, while RF performed the best for clay and sand. The lowest R^2^ values were consistently produced by the RT method, with some RT analyses not converging to a solution. In addition to the six soil properties where it gave the best results, PLSR was the second-best performing for four other properties (SOC, TN, clay, and silt) where R^2^ with PLSR was within 10% of the best method. Therefore, PLSR was selected as the most robust calibration method with DECS, and was used for subsequent analysis.

### 3.4. Comparison Among Fields 

**Figure 3:**
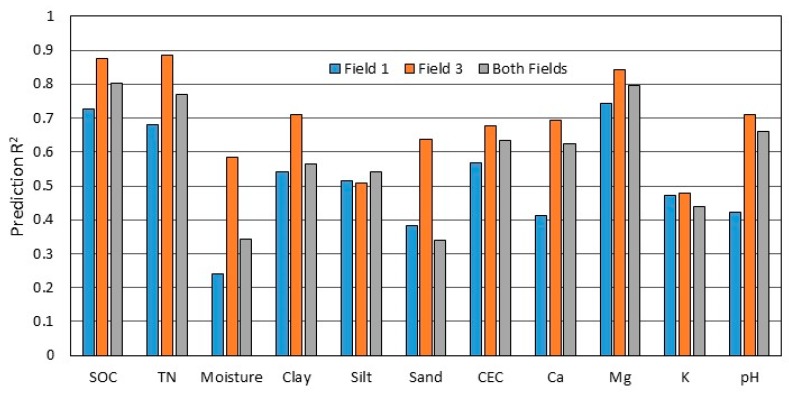
Prediction R^2^ values comparing results from Field 1, Field 3, and the combination of both fields using partial least squares regression (PLSR) on the combined dataset including depth, EC_a_, CI and spectra (DECS).

To examine the performance of DECS and PLSR at the field scale, the data from F1 and F3 were analyzed independently and compared. Soils and conditions of the two fields were similar at the time of sampling and data collection, except F3 was tilled approximately one week prior to measurement and the surface soil was loose and somewhat drier. Mean soil moisture in the surface horizon was 20.6% for F1 and 18.7% for F3. The profile soil moisture of F1 varied from 18.5% to 22.7% with a median of 20.6%, while the profile soil moisture of F3 varied from 16.1% to 21.5% with a median of 18.7%. For all soil properties except silt, the estimation accuracy for F3 was better than for F1 (Figure 3). For most soil properties, the estimation accuracy for the dataset including both fields was intermediate to that of F1 and F3. Exceptions were for silt fraction and K, where accuracy for all three datasets was similar, and for sand fraction (Figure 3).

A comparison of prediction R^2^ and RMSE across all soil properties for F1, F3, and the combination of F1 and F3 is shown in Table 5. Generally, estimation accuracy was better for F3 than F1, or the combination of F1 and F3. For each field and the combination, some soil properties were estimated more accurately by DECS and others by spectra alone, although in general the differences were slight. The only relatively large changes in R^2^ (magnitude >0.05) were for TN, soil moisture, and Ca in F1, moisture, silt, sand, and Ca in F3, and Ca in the combination dataset. Averaged across all soil properties, there was an increase in R^2^ of 0.03 and 0.01 with DECS for F3 and the combination, respectively, while there was a decrease of 0.01 with DECS for F1. There was little consistency across the datasets as to whether DECS or spectra were better. A notable exception was for Ca, where DECS was considerably better than spectra alone in all three datasets, likely due to EC_a_ responding to the clay mineralogy. Smectitic clay minerals such as montmorillonite dominate in this region [60]. These expandable clays exhibit high surface area and charge characteristics that increase the holding capacity for positively charged ions such as calcium [61,62] and likely contribute to the EC_a_ response.

In general, we note that vertical and lateral spatial correlation, which was not accounted for in our analyses, may have caused overestimation of model performance. However, in a study like this, where the main objective is a comparison of methods (i.e., for modeling, preprocessing, and dataset selection), it seems likely that the results and conclusions regarding the outcome of the comparisons would be similar given that the same validation set selection approach was used consistently. On the other hand, if the goal was to develop robust calibrations suitable for application to unsampled locations, different sample selection approaches [59] might be recommended.

## 4. Conclusions

In this study, the Veris P4000 instrument was used to acquire in-situ profile DRS spectra, EC_a_, and CI simultaneously down to a 1 m depth. Soil core samples were obtained at the same locations for laboratory measurement of soil properties, including SOC, TN, moisture, clay, silt, sand, Ca, Mg, K, CEC, and pH. Soil property estimates obtained using a variety of datasets, spectral preprocessing techniques, and modeling methods were compared. Conclusions from this research were:

- Of the preprocessing techniques investigated, absorbance spectra smoothed with a 30-point Gaussian window produced the most consistently accurate estimates, but only slightly better than absorbance spectra with a SNV transformation. When averaged across all soil properties, there was little difference in accuracy (ΔR^2^ = 0.03) among the 10 preprocessing techniques.
- Spectra alone provided better estimates of some soil properties while the multiple sensor (DECS) dataset performed better for others. However, DECS estimates improved by more than 5% in RMSE only for Ca, a marginal improvement with the additional complexity of multiple sensors.
- Overall, PLSR was the best modeling method, providing most accurate results for six soil properties and second best for another four, out of the 11 properties investigated. Estimation accuracy was more strongly affected by choice of modeling method than by choice of sensor dataset or preprocessing method.
- Accuracy varied considerably between two fields with similar soils, suggesting that in this case field-specific characteristics or management activities may have influenced the relationship of sensor data to soil properties.

The findings of this research regarding the best combination of sensors, preprocessing, and modeling techniques for in-situ estimation of profile soil properties should be confirmed through additional data collection and analysis for a wide range of soils and locations. Overall, this study showed that multiple soil physical and chemical properties could be estimated with good accuracy using profile spectral or DECS data with PLSR, demonstrating the ability of in-situ data collection to provide rapid assessment of soil properties at high spatial and/or temporal resolution for precision agriculture applications. 

## Figures and Tables

**Table 1:** Summary statistics of lab-determined soil properties. Coefficient of variation (CV) is in %.

Soil Property	Field 1				Field 3				Combination			
	Mean	SD ^†^	Range	CV	Mean	SD	Range	CV	Mean	SD	Range	CV
Samples from all soil horizons to 1.2 m profile depth (n = 148)												
SOC (%)	0.69	0.40	1.29	57.3	0.74	0.48	1.59	64.6	0.71	0.43	1.61	60.5
TN (%)	0.07	0.04	0.12	54.6	0.07	0.04	0.13	64.4	0.07	0.04	0.13	58.4
Moisture (%)	22.2	2.7	12.8	12.2	21.0	2.8	12.5	13.4	21.8	2.8	13.0	12.9
Clay fraction (%)	35.8	14.2	47.1	39.5	33.1	11.0	43.7	33.3	34.7	13.0	47.1	37.4
Silt fraction (%)	60.6	12.5	46.5	20.6	60.9	9.1	40.0	14.9	60.7	11.2	46.5	18.4
Sand fraction (%)	3.6	3.2	15.0	88.4	6.0	4.7	17.3	77.6	4.6	4.0	17.8	87.9
CEC (cmol·kg^−1^)	28.2	9.2	31.7	32.5	28.0	8.2	36.6	29.4	28.1	8.8	36.6	31.2
Ca (cmol·kg^−1^)	10.6	3.4	18.2	31.8	14.0	3.9	19.5	28.0	12.0	4.0	21.3	33.2
Mg (cmol·kg^−1^)	3.74	1.99	6.90	53.3	4.65	2.38	7.20	51.3	4.11	2.20	7.20	53.5
K (cmol·kg^−1^)	0.41	0.17	0.80	41.8	0.40	0.14	0.60	35.4	0.41	0.16	0.80	39.3
pH	4.36	0.63	3.20	14.5	5.19	0.70	2.80	13.6	4.70	0.78	3.20	16.5
Samples from surface horizon. Depth varied from 8 to 35.7 cm with a median of 21.8 cm (n = 33)												
SOC (%)	1.23	0.13	0.43	10.3	1.44	0.18	0.59	12.6	1.31	0.18	0.75	13.8
TN (%)	0.12	0.01	0.05	11.1	0.13	0.01	0.04	10.0	0.12	0.01	0.05	11.8
Moisture (%)	20.6	1.27	4.2	6.2	18.7	1.9	5.36	10.1	19.83	1.78	6.56	9.0
Clay fraction (%)	20.1	4.5	15.8	22.2	22.7	3.8	14.1	16.6	21.2	4.3	17.4	20.5
Silt fraction (%)	73.8	5.9	21.1	8.0	69.6	4.2	13.6	6.1	72.1	5.6	22.5	7.8
Sand fraction (%)	6.1	3.0	10.8	49.7	7.7	1.6	5.10	20.8	6.7	2.6	10.8	39.1
CEC (cmol·kg^−1^)	18.7	3.4	12.3	18.2	22.1	2.8	11.1	12.7	20.1	3.6	15.5	17.7
Ca (cmol·kg^−1^)	9.6	4.0	17.9	41.1	15.0	2.17	8.0	14.5	11.8	4.3	17.9	36.0
Mg (cmol·kg^−1^)	1.55	0.68	2.80	43.7	2.14	0.71	2.30	33.0	1.79	0.74	2.80	41.2
K (cmol·kg^−1^)	0.25	0.08	0.20	30.6	0.44	0.13	0.40	30.2	0.33	0.14	0.50	41.9
pH	5.16	0.76	2.90	14.7	6.22	0.32	1.10	5.1	5.59	0.81	2.90	14.4

^†^ SD = standard deviation, CV = coefficient of variation, SOC = soil organic carbon, TN = total nitrogen, CEC = cation exchange capacity, Ca = calcium, Mg = magnesium, K = potassium.

**Table 2:** Prediction R^2^ summary statistics for models using different spectral preprocessing techniques and calculated with partial least squares regression (PLSR) applied to the combined dataset including depth, EC_a_, CI, and spectra (DECS) for the individual fields and the combined field dataset. For each preprocessing technique and dataset, R^2^ statistics were across models for all soil properties. Grand mean R^2^ is the mean of the three datasets. Coefficient of variation (CV) is in %.

Preprocessing Technique	Field 1			Field 3			Combination (F1 + F3)			Grand Mean R^2^
	Mean	SD ^†^ & Range	CV	Mean	SD & Range	CV	Mean	SD & Range	CV	
Reflectance	0.51	0.14 0.44	28.1	0.65	0.16 0.51	24.9	0.57	0.15 0.48	25.3	0.58
Absorbance	0.52	0.14 0.43	26.8	0.67	0.15 0.49	23.1	0.59	0.16 0.49	27.4	0.59
Normalize + 9-point m.a.	0.51	0.14 0.45	28.2	0.65	0.18 0.54	27.3	0.61	0.20 0.70	32.6	0.59
9-point m.a. then normalize	0.52	0.13 0.39	25.8	0.62	0.18 0.55	28.4	0.59	0.14 0.45	24.1	0.58
30-point m.a.	0.53	0.13 0.38	24.6	0.68	0.14 0.44	21.0	0.59	0.16 0.50	27.9	0.60
30-point Lowess smoothing	0.52	0.15 0.50	28.8	0.65	0.16 0.48	25.3	0.60	0.16 0.47	25.8	0.59
30-point Gaussian smoothing	0.51	0.15 0.53	29.8	0.71	0.12 0.40	16.9	0.60	0.16 0.47	26.0	0.61
30-point exponential smoothing	0.52	0.13 0.42	25.7	0.64	0.17 0.47	26.2	0.60	0.15 0.45	24.2	0.59
SNV (standard normal variate)	0.52	0.16 0.42	29.9	0.68	0.14 0.43	27.4	0.61	0.14 0.44	23.7	0.60
SNV + 30-pt Gaussian smoothing	0.54	0.15 0.39	27.4	0.68	0.14 0.44	20.2	0.61	0.15 0.46	24.0	0.61

^†^ SD = standard deviation, CV = coefficient of variation, m.a. = moving average.

**Table 3:** Fit statistics for soil property estimation with each preprocessing technique, calculated with partial least squares regression (PLSR) and cross-validation on the combined dataset including depth, EC_a_, CI, and spectra (DECS). For each cell, the top row is R^2^ and the bottom row is root mean square error (RMSE; see Table 1 for units). Bold entries denote the highest R^2^ for each soil property, while underlined entries are the two lowest for each property.

Preprocessing Technique	SOC ^†^	TN	Moisture	Clay	Silt	Sand	CEC	Ca	Mg	K	pH
Reflectance	0.78 0.204	0.76 0.0201	0.43 2.103	0.60 8.264	0.54 7.638	0.31 3.360	0.60 5.560	0.49 2.839	0.79 1.005	0.48 0.117	0.57 0.506
Absorbance	0.79 0.198	0.77 0.0198	0.33 2.312	0.57 8.521	**0.56** 7.441	0.32 3.347	**0.65** 5.184	0.62 2.431	**0.81** 0.978	0.48 0.116	0.66 0.456
Normalize then 9-point m.a.	0.79 0.195	0.76 0.0199	0.38 2.205	0.57 8.493	0.45 8.265	0.29 3.411	0.61 5.508	0.62 2.453	0.80 0.982	0.47 0.118	0.63 0.476
9-point m.a. then normalize	0.80 0.194	0.76 0.0198	0.39 2.202	0.55 8.755	0.53 7.654	0.35 3.267	0.58 5.688	0.61 2.485	0.76 1.081	**0.49** 0.116	0.65 0.462
30-point m.a.	0.79 0.199	0.76 0.0198	0.31 2.333	0.61 8.130	0.51 7.825	0.34 3.282	0.64 5.261	0.63 2.438	**0.81** 0.977	0.43 0.122	0.62 0.479
30-point Lowess smoothing	0.80 0.196	0.76 0.0197	0.36 2.439	0.59 8.425	**0.56** 7.429	0.34 3.274	0.63 5.300	**0.64** 2.414	**0.81** 0.958	0.44 0.120	0.65 0.462
30-point Gaussian smoothing	0.80 0.194	0.77 0.0196	0.37 2.240	0.58 8.421	0.52 7.774	0.34 3.265	**0.65** 5.246	**0.64** 2.424	**0.81** 0.961	0.46 0.117	**0.67** 0.450
30-point exponential smoothing	0.79 0.198	0.76 0.0200	0.40 2.180	0.61 8.147	0.54 7.579	0.35 3.270	0.62 5.379	0.63 2.461	0.80 0.985	0.45 0.119	0.65 0.463
SNV (standard normal variate)	**0.81** 0.188	**0.78** 0.0193	**0.48** 2.020	0.62 8.000	0.55 7.544	**0.37** 3.230	0.63 5.386	0.58 2.594	0.79 1.008	0.45 0.118	0.65 0.457
SNV + 30-pt Gaussian smoothing	**0.81** 0.186	0.77 0.0196	**0.48** 2.040	**0.63** 7.868	0.54 7.556	0.35 3.248	0.61 5.479	0.63 2.426	0.78 1.020	0.44 0.119	0.66 0.460

^†^ SOC = soil organic carbon, TN = total nitrogen, CEC = cation exchange capacity, Ca = calcium, Mg = magnesium, K = potassium, m.a. = moving average.

**Table 4:** Fit statistics for soil property estimation with spectra alone, or the combination of depth, electrical conductivity, cone index, and spectra (DECS), calculated with partial least squares regression (PLSR) and cross-validation on the combined Field 1 and Field 3 dataset. For each cell, the top row is R^2^ and the bottom row is root mean square error (RMSE; see Table 1 for units).

	SOC ^†^	TN	Moisture	Clay	Silt	Sand	CEC	Ca	Mg	K	pH
Spectra	0.80	0.78	0.39	0.61	0.54	0.27	0.60	0.52	0.79	0.45	0.66
	0.193	0.019	2.218	8.089	7.626	3.468	5.536	2.795	1.011	0.118	0.453
DECS	0.80	0.77	0.34	0.57	0.54	0.34	0.63	0.62	0.80	0.44	0.66
	0.193	0.020	2.281	8.551	7.557	3.297	5.337	2.439	0.989	0.121	0.453

^†^ SOC = soil organic carbon, TN = total nitrogen, CEC = cation exchange capacity, Ca = calcium, Mg = magnesium, K = potassium.

**Table 5:** Prediction R^2^ and root mean square error (RMSE) calculated with different field data using the combination of depth, EC_a_, CI and spectra (DECS) and spectra alone. See Table 1 for units of RMSE. For each soil property within each field or the combination of both fields, bold text highlights the best R^2^ and RMSE.

	Field 1				Field 3				Combination			
Soil Property	DECS		Spectra		DECS		Spectra		DECS		Spectra	
	R^2^	RMSE	R^2^	RMSE	R^2^	RMSE	R^2^	RMSE	R^2^	RMSE	R^2^	RMSE
SOC ^†^	0.73	0.208	**0.77**	**0.194**	**0.87**	**0.173**	**0.87**	0.177	**0.80**	**0.193**	**0.80**	**0.193**
TN	0.68	0.022	**0.73**	**0.020**	**0.89**	**0.015**	0.85	0.017	0.77	0.020	**0.78**	**0.019**
Moisture	0.24	2.387	**0.34**	**2.210**	**0.59**	**1.838**	0.49	1.979	0.34	2.281	**0.39**	**2.218**
Clay	0.54	9.687	**0.55**	**9.463**	0.71	6.007	**0.72**	**5.834**	0.57	8.551	**0.61**	**8.089**
Silt	0.52	8.627	**0.53**	**8.647**	0.51	6.377	**0.60**	**5.802**	**0.54**	**7.557**	**0.54**	7.626
Sand	**0.38**	**2.502**	**0.38**	2.516	0.64	2.862	**0.74**	**2.453**	**0.34**	**3.297**	0.27	3.468
CEC	**0.57**	**6.055**	0.55	6.157	0.68	4.682	**0.70**	**4.625**	**0.63**	**5.337**	0.60	5.536
Ca	**0.41**	**2.587**	0.26	2.920	**0.70**	**2.232**	0.55	2.642	**0.63**	**2.439**	0.52	2.795
Mg	0.74	1.019	**0.75**	**1.003**	**0.84**	**0.959**	0.80	1.066	**0.80**	**0.989**	0.79	1.011
K	**0.47**	**0.127**	0.46	0.129	**0.48**	**0.103**	0.28	0.120	0.44	0.121	**0.45**	**0.118**
pH	0.42	0.487	**0.45**	**0.470**	**0.71**	**0.381**	0.71	0.386	**0.66**	**0.453**	**0.66**	**0.453**

^†^ SOC = soil organic carbon, TN = total nitrogen, CEC = cation exchange capacity, Ca = calcium, Mg = magnesium, K = potassium.
